# Dark Proteome Database: Studies on Dark Proteins

**DOI:** 10.3390/ht8020008

**Published:** 2019-03-27

**Authors:** Nelson Perdigão, Agostinho Rosa

**Affiliations:** 1 Instituto Superior Técnico, Universidade de Lisboa, 1049-001 Lisbon, Portugal; acrosa@isr.tecnico.ulisboa.pt; 2 Instituto de Sistemas e Robótica, 1049-001 Lisbon, Portugal

**Keywords:** dark proteome, molecular structure, homology modelling

## Abstract

The dark proteome, as we define it, is the part of the proteome where 3D structure has not been observed either by homology modeling or by experimental characterization in the protein universe. From the 550.116 proteins available in Swiss-Prot (as of July 2016), 43.2% of the eukarya universe and 49.2% of the virus universe are part of the dark proteome. In bacteria and archaea, the percentage of the dark proteome presence is significantly less, at 12.6% and 13.3% respectively. In this work, we present a necessary step to complete the dark proteome picture by introducing the map of the dark proteome in the human and in other model organisms of special importance to mankind. The most significant result is that around 40% to 50% of the proteome of these organisms are still in the dark, where the higher percentages belong to higher eukaryotes (mouse and human organisms). Due to the amount of darkness present in the human organism being more than 50%, deeper studies were made, including the identification of ‘dark’ genes that are responsible for the production of so-called dark proteins, as well as the identification of the ‘dark’ tissues where dark proteins are over represented, namely, the heart, cervical mucosa, and natural killer cells. This is a step forward in the direction of gaining a deeper knowledge of the human dark proteome.

## 1. Introduction

Many key insights and discoveries in the life sciences have been derived from atomic-scale 3D structures of proteins. Thanks to steady improvements in experimental structure determination methods, the PDB, or Protein Data Bank [1], which stores these structures recently went past 125,000 entries—a landmark in our understanding of the molecular processes of life. This still lags far behind the growth of protein sequence information, with less than 0.1% of UniProt [2] proteins linked to a PDB [1] structure. However, the understanding that evolution conserves structure more than sequence has led to the large-scale computation of structural model efforts [3]. Previously, we contributed to Aquaria [4], a source built upon a systematic all-against-all comparison of Swiss-Prot [2] and PDB sequences, resulting in a large number of template models [4]; this provides a depth of sequence-to-structure information currently not available from other resources to the visible proteome. However, there is a dark side of the proteome which we were the first to categorize [5], i.e., regions of protein sequences, or whole sequences, still remain inaccessible to either experimental structure determination or modelling approaches, such as Aquaria and others. This knowledge was updated and is kept in the independent Dark Proteome Database (DPD) [6] as a synonym for the structurally unknown part of the protein sequence universe, i.e., full sequences and/or regions of sequences for which the structure is currently undetermined [5,6]. The intrinsically disordered proteins (IDP) and/or regions (IDR), and more recently also dark proteome [7] are intrinsically unstructured proteins and/or regions, where their structure determination by conventional methods such as X-ray, nuclear magnetic resonance (NMR) crystallography and electron microscopy (EM) are arduous, due to the failure of homology methods, but most important due to their restless nature. Besides the fact that both definitions are focused on the structurally undetermined protein sequences, the first definition is broader and wider while the later narrows it to a small part of the former, i.e., only to the intrinsically unstructured regions. Nevertheless, both research paths stress the importance of studying the dark proteome, since it is still largely incomprehensible.

The present study serves as an introduction for two new features that were added to the Dark Proteome Database: (i) The availability of the ‘Features’ (FT) field of Swiss-Prot to deepen the knowledge of dark and non-dark proteins that allow the characterization of life domains and model organisms to get more thorough analysis; and (ii) the availability of an autonomy value per protein, allowing the analysis of the level of autonomy of dark and non-dark proteomes of those organisms as a whole. 

Therefore, the main goals of this work are: (i) To detail even more the dark proteins present in the four domains of life, model organisms, and Homo Sapiens and (ii) to map how much of darkness is still present in these model organisms, since they are so important for mankind, i.e., to how much we do and do not know about a certain organism just by looking at the amount of darkness that it holds? Due to the number of existing organisms, we had to select the most important ‘model’ organisms (for us humans) while the ones remaining are work in progress. This choice is not innocent, since sequences originated from non-model organisms are known by the lack of sequence annotation, without being true orphan sequences. In the DPD [6] we started with information from Homo Sapiens because it is of direct concern to us, but Earth is the home of millions of organisms and only approximately two handfuls of them were adopted as “model” for biological experiments, concerning food, infirmities, diseases, and threats. In an ideal world, we would have access to all the knowledge concerning every single organism that inhabits this planet. Since, we are not in the ideal world, these models were selected according to our present reality. In short, studying all of them in depth with today’s technology is almost impossible because experimentation is complex, time consuming, and very expensive.

Keeping our reality in mind, we have to remember that some of the most valuable methods in biological research are invasive or require an organism’s death. For all the previous reasons and more, much of this work is impossible or unethical to perform on humans and in certain organisms. As a solution, biologists have selected some model organisms to be used as testers. The following list contains a simple plant, a worm, a bacterium or prokaryotic, a simple eukaryotic, and a complex eukaryotic or mammal. 

Finally, we will analyze the human organism (Homo Sapiens) in much more detail, including the genes from where dark proteins came from (‘dark’ genes) and tissues where dark proteins are expressed (‘dark’ tissues).

**Arabidopsis thaliana (Plant)**

Arabidopsis thaliana is considered a weed, also known as mouse-ear cress and is the most widely used plant as a model organism. One reason Arabidopsis makes a good model is because it undergoes the same exact processes of growth, flowering, and reproduction as most complex plants, taking only about one month and a half to grow completely producing a huge quantity of seeds in the process. Another reason is due to the fact that Arabidopsis has one of the smallest genomes in the plant kingdom with only 135 mega base pairs and five diploid chromosomes. It is the first plant with a completely sequenced genome.

**Caenorhabditis Elegans (Worm)**

Caenorhabditis Elegans is a soil worm or nematode and is considered a model for multicellular organisms. The reason why it is such a good model is related to the fact that it shares a common ancestor (“urbilaterian ancestor”) with humans that lived 500 million years ago, therefore sharing most of the genes that govern most modern organismal development and disease, such as the human and nematode. This is extremely important because many genetic and developmental experiments are impossible in humans (technically and ethically). Some evasive procedures associated with experiments are extremely time-consuming or cost-intensive. C. Elegans presents itself as a reliable alternative because of its short generation time (four days), and due to its complete anatomy also being known. The adult hermaphrodite has exactly 959 cells, while the adult male has exactly 1031 cells and both are transparent. This allows researchers to relate behaviors to particular cells, to trace the effects of genetic mutations and gain relevant insights into the mechanisms of development and ageing. Therefore, due to the evolutionary conservation of gene function, C. Elegans is the ideal model organism to trace basic genetic mechanisms of human development and disease, such as cancer and neurodegenerative diseases. C. Elegans was the first multicellular eukaryotic organism to have its whole genome sequenced. 

**Escherichia Coli (Bacteria)**

The Escherichia Coli (E. Coli) can be found in the intestine of warm blood organisms. The reasons why this bacterium is such a good model are firstly, because it is a simple organism, and secondly that it can be cultured and grown easily and inexpensively in a laboratory. However, the main reason why it is heavily tested and trialed even though a bacterium, is related to the fact that its basic biochemical mechanisms are common to the human organism. Another important aspect is that E. Coli was the reason for first understanding the transcription factors that activate and deactivate genes in the presence of a virus. From that point on, E. Coli was used as a host in genetic engineering and especially in health, producing several types of proteins, encoding them with the majority of human genes that are applied as medicinal drugs. There are several variants of E. Coli, most of them harmless. However, some of them can be lethal and are responsible for product recall due to food contamination or food poisoning. This bacterium was the first prokaryotic model organism to have its genome sequenced (K12 strain) and has a single circular chromosome with 4.6 million base pairs.

**Saccharomyces cerevisiae (Yeast)**

Saccharomyces cerevisiae is recognized as the key factor in brewing for centuries. The main reasons for it being considered a model organism are related to its easy culture, fast growing process, and inexpensive production in a laboratory. Being a eukaryote, it share the same complex internal cell structure of plants or animals without the high percentage of junk DNA present in more complex eukaryotes facilitating research. Since S. cerevisiae is biochemically very similar to the human organism, many studies about the molecular processes involved in cell cycle, meiosis, recombination, DNA reparation, ageing, and other fundamental areas of biology were possible. S. cerevisiae was the first eukaryotic genome to be completely sequenced. It is composed of 12 chromosomes containing approximately 12.2 million base pairs.

**Mus musculus (Mouse)**

The Mus Musculus (Mouse) is the most famous model organism because is the mostly used mammal in medicine and biology scientific communities. The main reason why is such a good model is that it is a mammal and, therefore, has organs and development processes that are very similar to the human organism; next, they are easily reproductible, grow fast, and very easy to maintain and manipulate in a laboratory; finally, mice suffer from most of the diseases and calamities that affect mankind. Therefore, mice have an extremely important role in the development of new pharmaceuticals for humans. The mouse genome consists of 40 chromosomes with 2.63 billion amino acids. 

In our previous work [5], we analyzed the four domains of life where the conclusions were: The dark proteome is mostly not disordered, mostly not compositionally biased, mostly not transmembrane, but more important and unexpectedly, it is mostly “Unknown Unknowns” [5]. The dark protein portion of “Unknown Unknowns” in eukaryota is almost 50%. It is composed of ordered, globular, and low compositional bias proteins. In the case of bacteria this percentage is over 50% and in case of archaea reached almost 70%. Finally, in viruses this percentage reached almost 75% [5]. There were several questions raised at that time, that are still valid today, such as: Could we detail more dark proteins location and environment? Could we detail even more its functions? 

## 2. Materials and Methods

*Dataset*: The set of protein sequences selected for this work were prevenient from Swiss-Prot release of July 2016 [6]. The protein structures were extracted from PDB on July 2016. Predictions from PSSH2 [4], PMP [8] and Predict Protein [9] are versions from July 2016. Finally, Protein-Protein Interaction (PPI) information is prevenient from STRING [10], also from July 2016. The Swiss-Prot dataset is composed of 550.116 proteins and divided in four kingdoms: 19.370 protein sequences from archaea, 332.327 from bacteria, 181.814 from eukaryota and 16.605 from viruses. The number of proteins sequences for each model organism are: 14.349 protein sequences for Arabidopsis, 3.652 for C. Elegans, 669 for E. Coli, 42 for S. cerevisiae, 16.747 for Mouse and finally 20.209 for Human.

*Mapping Darkness*: For each Swiss-Prot protein, each residue was categorized as “non-dark” if it met either one of the following criteria: If the residue was aligned onto the “ATOM” record of any PDB entry [1] in the corresponding Aquaria matching structures entry (criterion A); or if the residue was aligned onto a PDB entry in the corresponding UniProt entry (criterion B). All other residues were categorized as “dark.” We then calculated a “darkness” score (*D*) as defined in Reference [5]. If *D* = 0 this means it is PDB or a white protein, otherwise, if *D* = 1, this means it is a dark protein. If 0 < *D* < 1 means it is a grey protein with grey regions containing dark regions [5].

**Figure 1:**
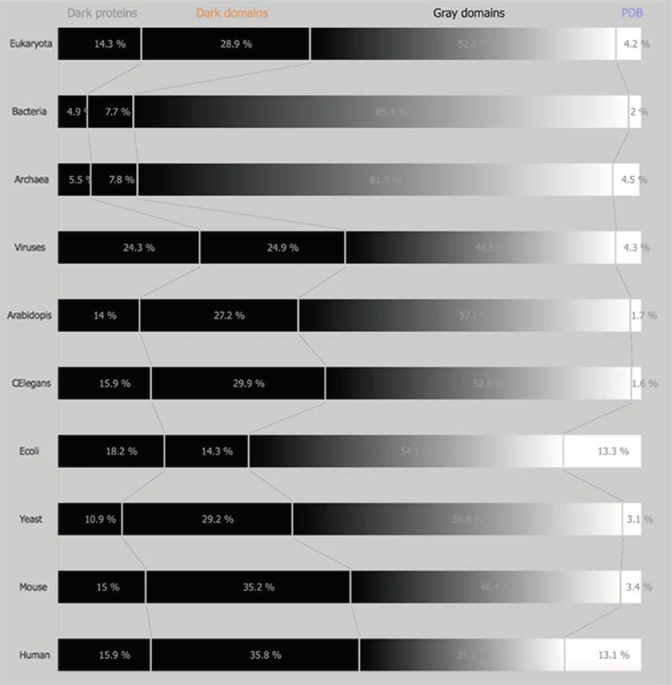
Darkness map *per* life domain and *per* model organism.

**Figure 2:**
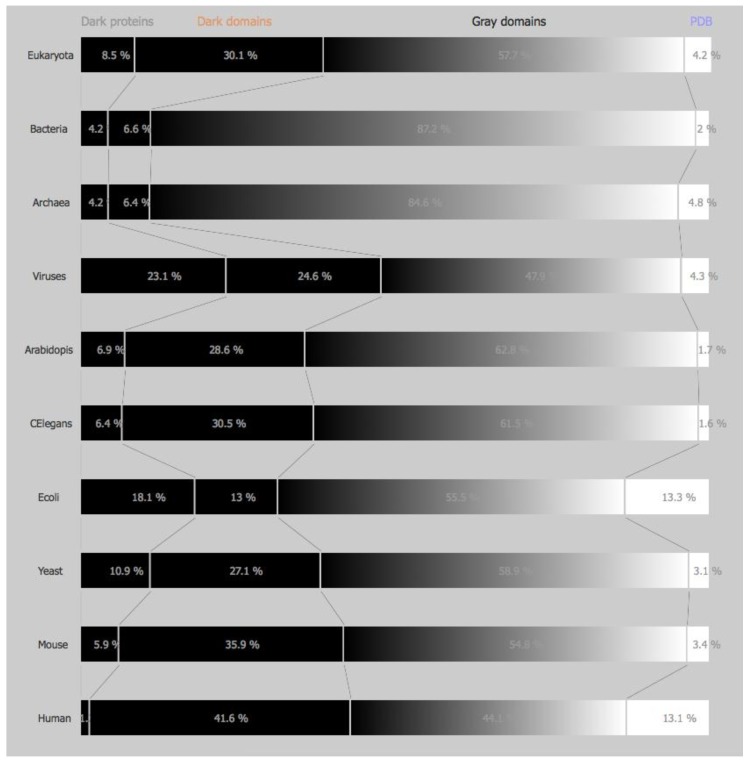
Darkness map *per* life domain and *per* model organism with PMP.

*Dark and non-Dark Percentages*: The percentages displayed for “dark” proteins, “dark” regions, grey regions, and PDB regions present in the above sets (domains of life and model organisms) consist first in obtaining both “dark” and PDB proteins in the sets mentioned above. Next, “dark” as well as, “non-dark” regions are mapped, subtracting the “dark” proteins from the former, and subtracting the PDB proteins from the later, obtaining the cardinality of “dark” regions and “non-dark” regions. If we divide the above cardinalities by the total amount of dark and non-dark regions, we obtain the percentages presented in Figure 1 and Figure 2.

*Annotation Enrichment*: The functional analysis compares annotations between dark and non-dark proteins in a reliable manner, by the application of annotation enrichment to the ‘Description’ (DE) field, which were now extended with the ‘Features’ (FT) field of the Swiss-Prot proteins through Fisher exact tests [11,12] followed by the Benjamini–Hochberg false discovery correction [13] with α, the fraction of false positives was considered acceptable, set to 1%, and accepted only annotations with an adjusted *p* value of ≤ 1%, calculated via:

(1) $$p^{adjusted}=\;Min\left[\frac{p*n}{k+1},\;1\right]$$

where *p* is from Fisher’s test, *n* is the total of number of annotations in the set, and *k* is the rank of the largest *p* value that satisfies the false discovery criteria as in Reference [6]. This approach was then repeatedly applied to compare dark and non-dark proteins across various sets of organisms.

**Figure 3:**
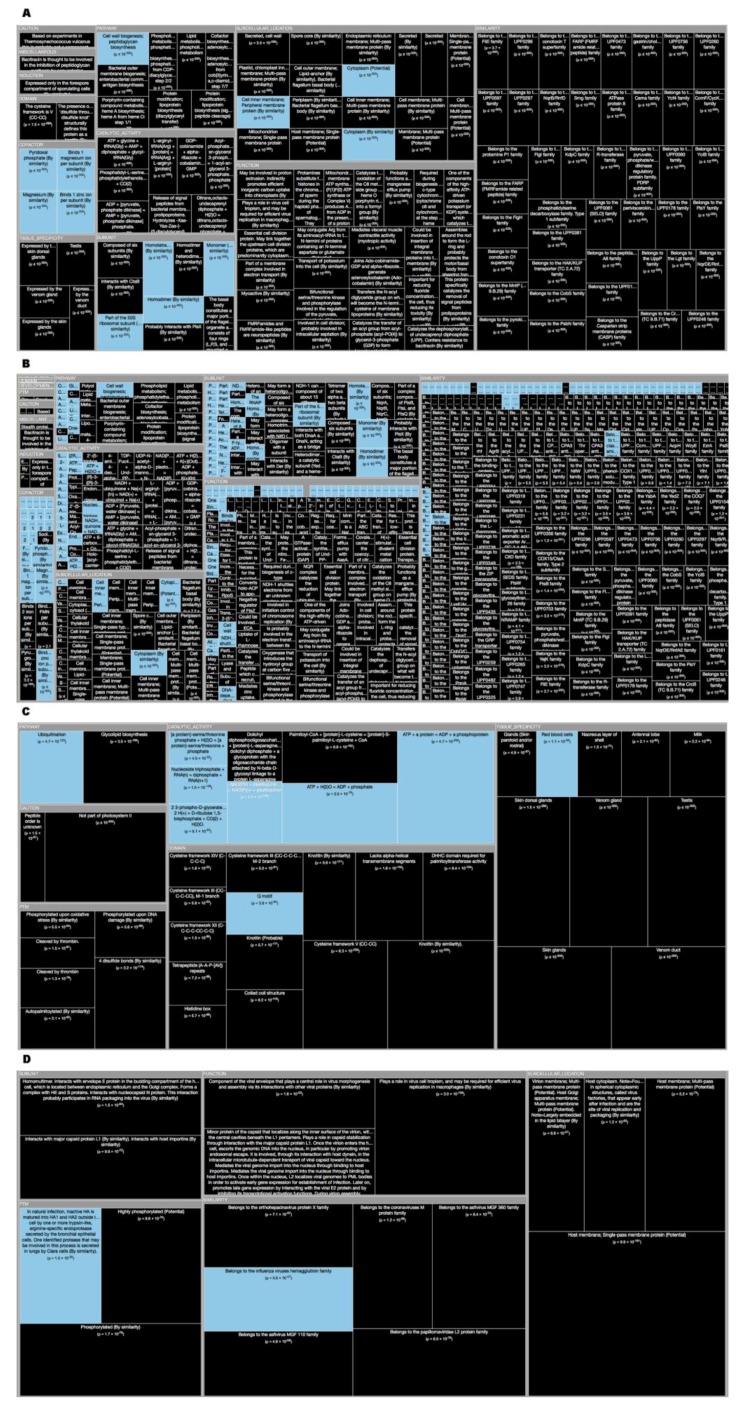
TreeMap showing all annotations (descriptions) over- and under-represented in dark proteins for all the proteins in Swiss-Prot divided by the four domains (details in Table 1 and dataset S1). Functional annotations over- or under-represented in dark proteins. Pooling annotations for all proteins, we used enrichment analysis to find biological functions associated with dark proteins. The tree map shows all over- and under-represented annotations (dark and blue, respectively) in 21 functional categories; cell area indicates annotation significance (scaled to –log10(*P*), using the adjusted *p* value from Fisher’s exact test – see methods). (**A**) Archaea; (**B**) Bacteria; (**C**) Eykaryota; (**D**) Viruses. A cut-off value (-log10(p)) = 50 was applied for figure readability.

**Figure 4:**
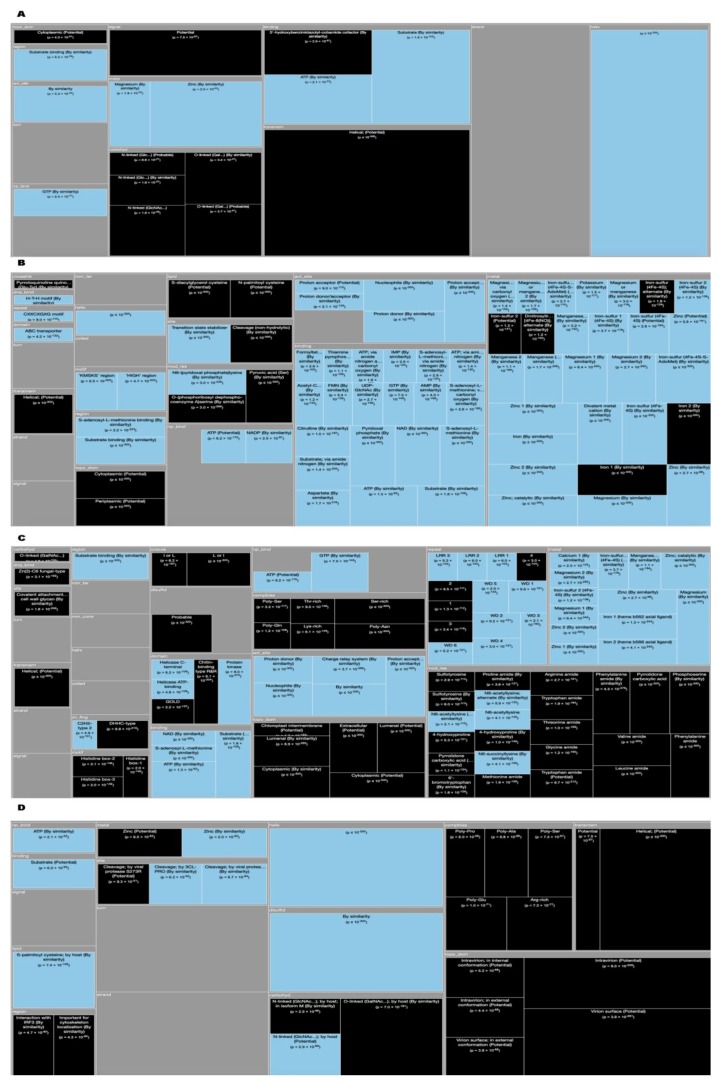
TreeMap showing all annotations (features) over- and under-represented in dark proteins for all the proteins in Swiss-Prot by the four domains of life and divided into 36 functional categories (details in Table 2 and dataset S2). (**A**) Archaea; (**B**) Bacteria; (**C**) Eykaryota; (**D**) Viruses. A cut-off value (-log10(p)) = 50 was applied for figure readability.

**Figure 5:**
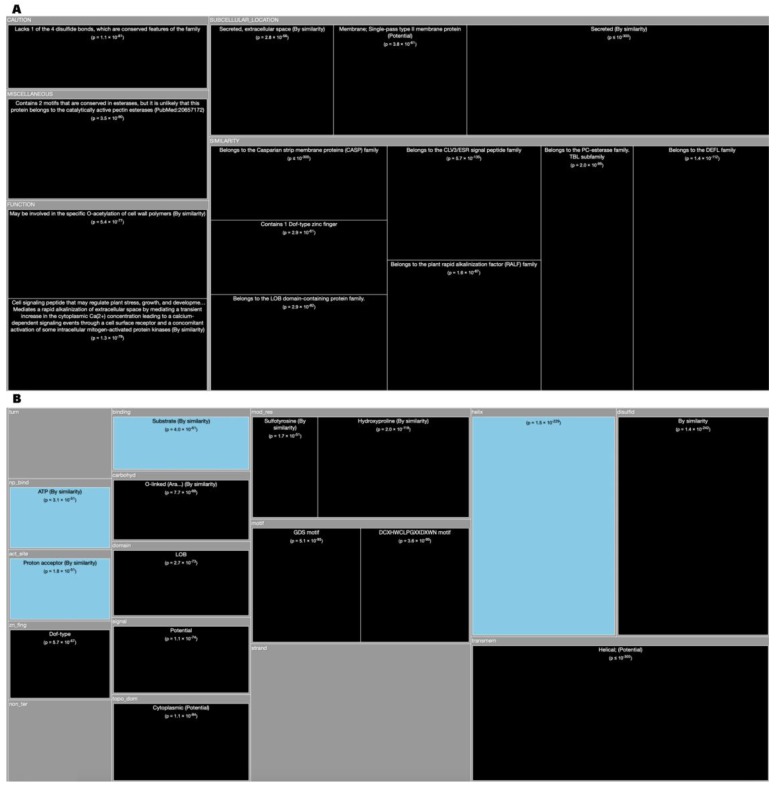
**(A)** TreeMap showing all annotations (descriptions) over-represented in dark proteins for organism Arabidopsis Thaliana (details in dataset S1); **(B)** TreeMap showing all annotations (features) over-represented in dark proteins for organism Arabidopsis Thaliana (details in dataset S2). A cut-off value (-log10(p)) = 50 was applied for figure readability.

**Figure 6:**
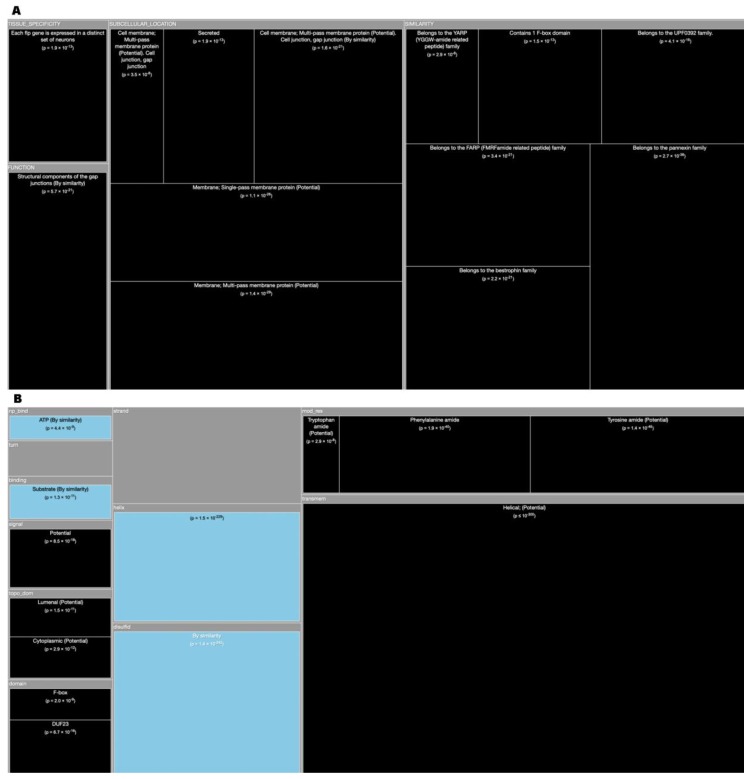
**(A)** TreeMap showing all annotations (descriptions) over- and under-represented in dark proteins for organism C. Elegans (details in dataset S1); **(B)** TreeMap showing all annotations (features) over- and under-represented in dark proteins for organism C. Elegans (details in dataset S2). A cut-off value (–log10(p)) = 10 was applied for figure readability.

**Figure 7:**
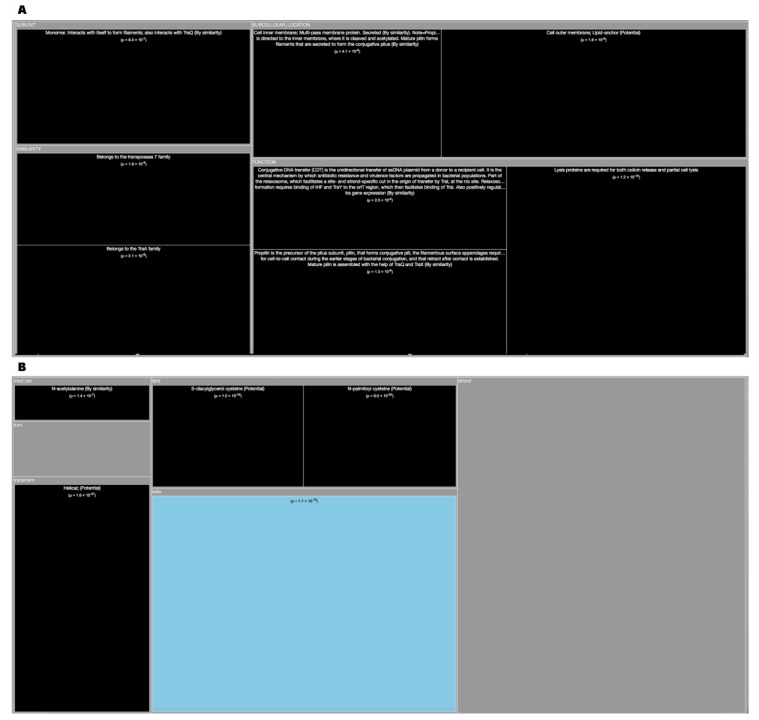
**(A)** TreeMap showing all annotations (descriptions) over-represented in dark proteins for organism E. Coli (details in dataset S1). **(B)** TreeMap showing all annotations (features) over-represented in dark proteins for organism E. Coli (details in dataset S2). A cut-off value (–log10(p)) = 0 was applied for figure readability.

**Figure 8:**
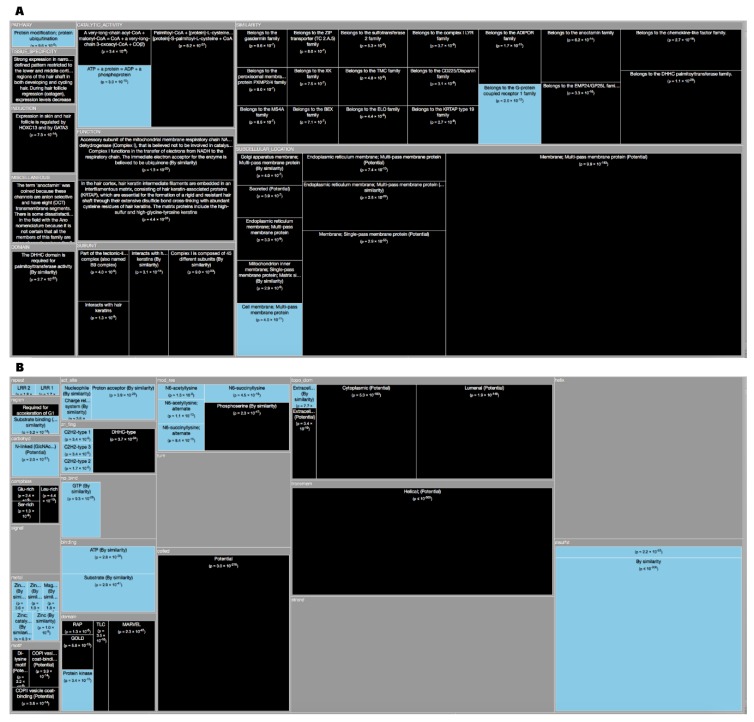
**(A)** TreeMap showing all annotations (descriptions) over-represented in dark proteins for organism Mus Musculus (details in dataset S1). **(B)** TreeMap showing all annotations (features) over-represented in dark proteins for organism Mus Musculus (details in dataset S2). A cut-off value (–log10(p)) = 0 was applied for figure readability.

**Figure 9:**
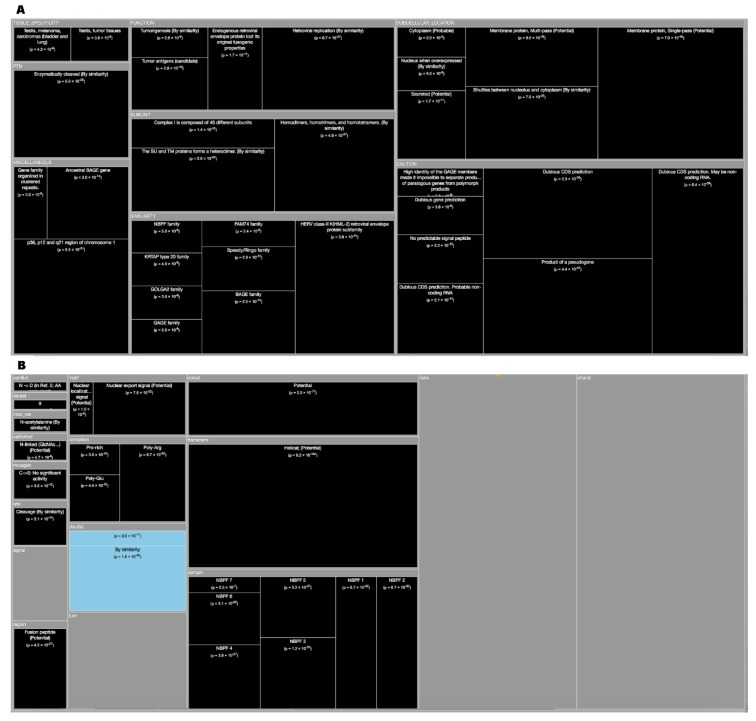
**(A)** TreeMap showing all annotations (descriptions) over-represented in dark proteins for organism Human (details in Table 3 and dataset S1). **(B)** TreeMap showing all annotations (features) over-represented in dark proteins for organism Human (details in Table 4 and dataset S2). A cut-off value (–log10(p)) = 0 was applied for figure readability.

*Tree Maps*. From the ‘Description’ enrichment analysis results, we selected 21 (of 25) subcategories judged to be most significative and visualized them using a tree map [14]. For the ‘Features’ enrichment analysis results we selected 36 (of 39) subcategories. The removed subcategories included those with relatively few results—or results with relatively high adjusted *p* values—as well as subcategories such as “Similarity,” which only give information about groups of very similar proteins and the specific functions they perform; although interesting, these specific annotations do not reveal more general properties of dark proteins. In Figure 3, Figure 4, Figure 5, Figure 6, Figure 7, Figure 8 and Figure 9 the results were displayed using the D3 zoomable tree map library (bost.ocks.org/mike/treemap); some annotation terms have also been reworded to improve readability.

*Mapping Autonomy per protein*. For every human protein we evaluated its autonomy, i.e., using STRING [10] we counted how many others it interacts with. The STRING scheme classifies its functional link confidence into three different scores [15]: Low (< 400), medium (400 < score < 700) and high (> 700) confidence scores measuring the confidence in the pair-wise functional interactions of the networks produced. Even assuming that sequence data is accurate, computational tools can introduce noise when generation sequence similarity data occurs. Taking this noise into account, it is suggested to set a cut-off score above which an interaction is highly probable. In terms of a functional classification accuracy, what matters is a high confidence score of 700 or higher [16], however, low and medium confidence were done for comparison purposes (results not shown). Therefore, for each Swiss-Prot protein, we categorized its autonomy as:

(2) $$Autonomy\;Score=\;\{\begin{array}{c} 1-\left(0.N\right)\;if\;m\left(N\right)=0\;and\;0\leq N\leq 900\\ \;\;\;\;\;\;\;\;\;0\;if\;m\left(N\right)\neq 0\;and\;N>900 \end{array}$$

where *m(N)* indicates the number of matches that occur for a link score of *N*. This means, if a protein has *m(0)* equals to zero matches, then the protein is fully autonomous because at the lowest quality cut-off score no interactions occur between it and other proteins. On the other hand, if at the highest cut-off score there still exist interactions with other proteins (i.e., *m(900)* is not zero) then it can be concluded that the protein is completely non-autonomous. 

*Dark Genes*. For each chromosome in Homo Sapiens, we then constructed a list of dark proteins sorted by the position of the central nucleotide of the corresponding gene, determined using UCSC assembly hg19 [17]. In some cases, due to gene duplication, multiple copies of the same dark protein were annotated as arising from multiple genes in the same chromosome; in such cases, we considered only the first occurrence, and removed all other copies from the list. For each chromosome, we then calculated the longest run of dark proteins, and assigned a *p* value by calculating how many times a run with the same number of dark proteins or more occurred by chance in 1,000 random re-orderings of proteins along the chromosome. Note, that the cluster results are very conservative where the chance of a false positive is 1/1000 on a per-chromosome basis; thus, there are probably more such ‘dark’ gene clusters.

*Dark Tissues*. Finally, we have used ProteomicsDB [18] that contains mass-spectrometry data from protein expression measurements from 16,857 liquid chromatography tandem-mass-spectrometry (LC-MS/MS) experiments involving human tissues, cell lines, body fluids including data from PTM studies, and affinity purifications. To obtain the normalized intensity values for each protein from ProteomicsDB, the protein expression API was used. These values measure the relative abundance of peptides of each protein in a specific sample in a logarithmic scale. As we did not find any mass-spectrometry data for 1,391 dark proteins and 2,762 non-dark proteins, we considered these empty entries as 0.

## 3. Results

### 3.1. Dark Proteome Database Status 

This work tries to answer the questions formulated in the introduction, starting by presenting the status of DPD [6] in July 2016 (Figure 1), i.e., the percentage of dark proteins, dark regions, grey regions, and PDB regions as defined in Reference [5] for the four domains of life plus the six model organisms described above. Using the more stringent definition of darkness as defined in Reference [5] we can observe the status of DPD (Figure 2) including the PMP (Protein Model Portal) [8] predictions for the same four domains of life plus the six model organisms.

Comparing the actual version of DPD without PMP (Figure 1) and with PMP (Figure 2), we can observe marginal differences either in the domains of life or in the model organisms studied in this work. Therefore, henceforth, this study will only focus on a DPD version without PMP. 

Comparing now the initial version of DPD [5] (Figure S1) with the current version of DPD [6] and starting by the domains of life we can observe that dark proteins (from: E:15.2%, B:5.3%, A:5.8% V:28.1% to: E:14.3%, B:4.9%, A:5.5%, V:24.3%) and dark regions (from: E:28.8%, B:8.2%, A:7.9%, V:26.3% to: E:28.9%, B:7.7%, A:7.8%, V:24.9%) percentages had decreased compared with the previous version of DPD (5), while grey proteins (from: E:52.1%, B:84.6%, A:81.8%, V:41.7% to: E:52.6%, B:85.4%, A:81.9%, V:46.5%) and white (PDB) regions (from: E:3.8%, B:1.8%, A:4.5%, V:3.9% to: E:4.2%, B:2.0%, A:4.8%, V:4.3%) the percentages had increased (Figure 1).

Focusing now on model organisms and performing exactly the same reasoning as the one above, we observe the same tendency (Figure S1), i.e., we can observe that dark proteins (from: Ar:13.6%, CE:17.2%, EC:17.8%, SC:12.7%, MM:15.4%, HS:16.7% to: Ar:14.0%, CE:15.9%, EC:18.2%, SC:10.9%, MM:15.0%, HS:15.9%) and dark regions (from: Ar:26.8%, CE:29.4%, EC:15.2%, SC:30.3%, MM:35.3%, HS:35.5% to: Ar:27.2%, CE:29.9%, EC:14.3%, SC:29.2%, MM:35.2%, HS:35.8%) percentages had decreased in general (except for Arabidopsis, E. Coli and Yeast) compared with the previous version of DPD [5], while grey proteins (from: Ar:57.9%, CE:51.0%, EC:54.4%, SC:53.8%, MM:46.2%, HS:36.2% to: Ar:57.2%, CE:52.6%, EC:54.1%, SC:56.8%, MM:46.4%, HS:35.2%) and white (PDB) regions (from: Ar:1.7%, CE:1.4%, EC:12.7%, SC:3.2%, MM:3.1%, HS:11.7% to: Ar:1.7%, CE:1.6%, EC:13.3%, SC:3.1%, MM:3.4%, HS:13.1%) the percentages had increased in general (Figure 1).

It can be concluded by looking at the previous results that the general knowledge concerning the four domains of life has increased since the number of dark regions and dark proteins percentages decreased, while the grey and white regions percentages increased. The previous conclusion could also apply in model organisms but not so straight, since there were dark regions and dark proteins areas that expanded, while grey and white areas shrank, even if marginally.

However, if we look at the overall picture (Figure 1), even with this increase in PDB and grey regions, we conclude that for the four domains of life the percentage of the dark proteome is still very high in eukaryotes (43.2%) and in viruses (49.2%) and very low in archaea (13.3%) and in bacteria (12.6%). Considering PMP, the scenario does not improve much, with 38.6% of darkness present in eukaryotes, 47.7% in viruses, 10.6% in archaea, and 10.8% in bacteria (Figure 2). 

Looking at the model organisms the view is not much different, with Ar:41.2%, CE:45.8% EC:32.5%, SC:40.1%, MM:50.2%, HS:51.7%, i.e., with around half of their proteome still in the dark (Figure 1). Considering PMP we obtain Ar:35.5%, CE:36.9% EC:31.1%, SC:38.0%, MM:41.8%, HS:42.8%, i.e., more than one third of each organism (except E. Coli) remains in the dark (Figure 2).

#### 3.1.1. Domains of Life

Going deeper we wanted to analyze and visualize these dark proteins by domains of life, and to do so we used TreeMaps to analyze Swiss-Prot fields ‘Description’ (DE) (Figure 3) and ‘Features’ (FT) (Figure 4) using “Annotation Enrichment” (See Methods) to point towards reliable conclusions.

Observing Swiss-Prot proteins through the TreeMap (with 21 functional categories) of the ‘Descriptions’ field (DE) by life domain show the following: 

Archaea dark proteins (Figure 3A) in “Subcellular Location” are over-represented in ‘Cell membrane’, ‘Cell inner membrane’ and being ‘Secreted’. Dark proteins are under-represented in ‘Cytoplasm’. Dark proteins in this domain of life are over-represented in “Tissue” like ‘Venom Gland’ and ‘Venom Duct’ as well as, in ‘Skin (including Dorsal) Glands’ and ‘Testis’, being under-represented in only two “Tissues”: ‘Red blood cells’, and ‘Ubiquitous’. Dark proteins were under-represented in many “Catalytic site” and “Pathway” annotations, where inference often requires similarity to a PDB structure. Dark proteins in Archaea organisms have several “Functions” that are ‘Responsible for cell division’, ‘Proton extrusion’, as well as ‘Transport of potassium’, among others. These and the following results can be verified at Reference [19] by applying the indicated cutoff values at the slider button.

Bacteria dark proteins (Figure 3B) like archaea are over-represented in “Subcellular Location” like ‘Cell membrane’, ‘Cell outer membrane’, ‘Cell inner membrane’, ‘Lipid anchors’ and being ‘Secreted’. Dark proteins are also under-represented in ‘Cytoplasm’. Dark proteins were under-represented in many “Catalytic site”, “Pathway” and “Subunit” (namely ‘Ribosomal’) annotations. Dark proteins in bacteria organisms have several “Functions” that are ‘Responsible for cell division’, ‘Transport of potassium’, as well as, being ‘Catalyzers’ and being involved in ‘Initiation control of chromosome replication’ among others.

Eukaryota dark proteins (Figure 3C) are over-represented in “Subcellular Location”, such as many specific secretory tissues and exterior environment, such as ‘Venom Gland’, ‘Venom Duct’, in ´Skin (including Dorsal) Glands’, ‘Testis’ and ‘Milk’ and under-represented in the same two “Tissues” annotations like archaea. Dark proteins were also over-represented in ‘Cysteine’ domains and ‘Disulfide bonds’. Additionally, eukaryotic dark proteins were over-represented in ‘Cleavage’ and other post-translational modifications known to prepare proteins for harsh environments. Dark proteins like archaea were under-represented in many “Catalytic activity” and “Pathway” annotations.

Finally, viruses dark proteins (Figure 3D) are over-represented in “Subcellular Location”, such as ‘Host Membrane’, ‘Host cytoplasm’, ‘Virion’ and ‘Virion membrane’. Dark Proteins are associated with “Functions” of ‘Viral infection’, ‘Virus transportation’, as well as, ’Replication’ on ‘Hosts’. Dark proteins were under-represented in many annotations like “Catalytic activity”, “Pathway” and “Enzyme regulation”.

We wanted to detail even more the Dark Proteome therefore, we used the ‘Features’ field (FT) which is a subsection of the ‘Description’ field (DE) by life domain through TreeMap (with 36 functional categories). Observing Figure 4, allow us to conclude the following: 

Archaea dark proteins (Figure 4A) are over-represented in “Transmembrane” as ‘Helical’ and in “Topological Domains”, ‘Extracellular’ and ‘Cytoplasmic’. They are also over-represented in “Carbohyd", “Compositional bias” of ‘Poly-Glu’ and in “Non-Standard” amino acids (Selenocysteine and Pyrrolysine). Dark proteins are under-represented in “Active Sites”, “Helixes”, “Metal”, “NP-bind”, and “Binding”. 

Bacteria dark proteins (Figure 4B) like archaea are over-represented in “Transmembrane” as ‘Helical’ and in “Topological Domains”, ‘Cytoplasmic’ and ‘Periplasmic’. They are also over-represented in “Lipid", "Crosslink” and “Compositional bias”. Dark proteins are under-represented in “Metal”, “Binding”, “Active Sites”, “Domain”, “Regions”, and “NP-bind”.

Eukaryota dark proteins (Figure 4C) are over-represented in “Topological Domains”, ‘Extracellular’, ‘Cytoplasmic’ and ‘Lumenal’. Dark proteins were also over-represented in “Compositional bias”, “Motifs”, and “Unsure”. Dark proteins are under-represented in many “Binding”, “Metal”, “NP-bind”, “CA-bind”, and “Crosslink” annotations.

Finally, in viruses, dark proteins (Figure 4D) are over-represented in “Transmembrane” and in “Topological Domains”, ‘Intravirion’ and ‘Virion Surface’. Dark Proteins are also over-represented in “Compositional bias”. Dark proteins were under-represented in many annotations like “Binding”, “Active Sites”, “Metal”, and “Sites”.

#### 3.1.2. Model Organisms

**Figure 10:**
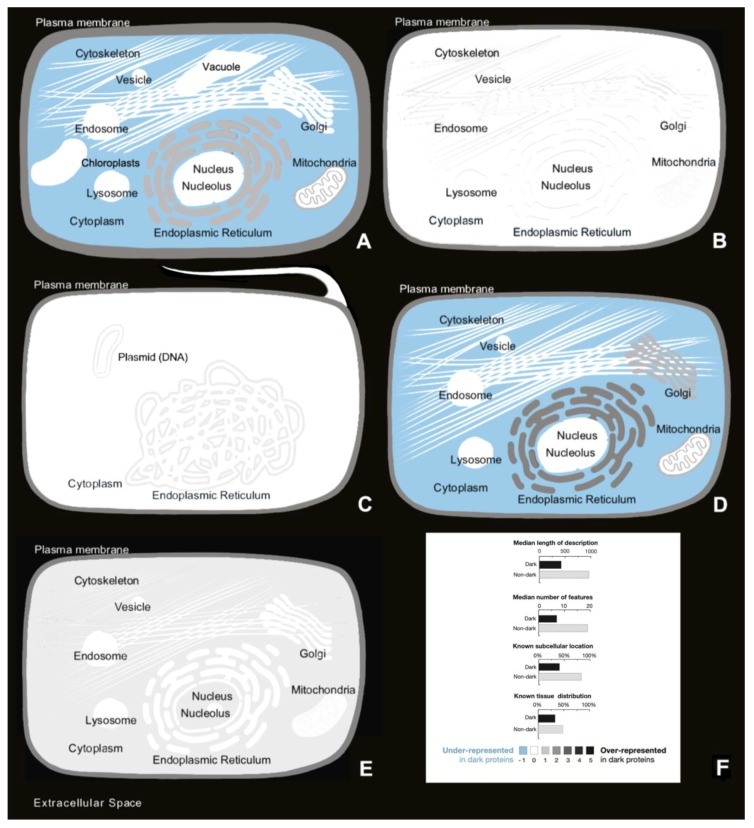
**(A)** Cellular locations over- and underrepresented of dark proteins in Arabidopsis; **(B)** Cellular locations over- and underrepresented of dark proteins in C. Elegans; **(C)** cellular locations over- and underrepresented of dark proteins in E. Coli; **(D)** cellular locations over- and underrepresented of dark proteins in Mouse; **(E)** cellular locations over- and underrepresented of dark proteins in Human; **(F)** dark proteins in Human have shorter functional descriptions, fewer sequence-specific features, and less complete annotation about subcellular location and tissue distribution.

Arabidopsis has 41.2% of its proteome in the dark (Figure 1). The conclusions that we can infer through the analysis of the corresponding TreeMap (Figure 5A) for ‘Descriptions’ (DE field of Swiss-Prot files) are: That dark proteins are over-represented in “Subcellular Location” such as ‘Endoplasmic reticulum membrane’ either with ‘Single-pass’ or ‘Multi-pass’. They are also ‘Secreted’ in the ‘Extracellular space’ or through the ‘Cell wall’. Dark proteins most evident “Functions” are related with ‘Transcription factors’ as well as with ‘Regulation of cell fate’, and even with ‘Regulation of the plant stress, growth and development’. Dark proteins are under-represented in ‘Chloroplast’ and in ‘Cytoplast’ (not shown). Analyzing the results of the corresponding TreeMap (Figure 5B) of ‘Features’ (FT field of Swiss-Prot files) we can observe that: The dark proteins are located mostly in the extension of “Transmembrane” regions where its “Topological domain” are ‘Cytoplasmic’ and ‘Extracellular’ (not shown). Dark proteins are common in “Signal” sequences (prepeptides) and related with transcription factors being also associated with “Disulfides”. They are under-represented in “Helix”, “Binding”, and “NP-bind” (Figure 10A).

The C. Elegans organism contains 45.8% of dark proteome (Figure 1). Analyzing again the TreeMap of ‘Descriptions’ (Figure 6A) we conclude that dark proteins are mostly located in the ‘Membrane’, especially in dark proteins with ‘Multi-pass’ or ‘Single-pass’ and ‘Cell membrane’. The main “Functions” of the dark proteins are “Structural on the gap junctions”, also present in “Neuropeptides”. Turning now our attention to the TreeMap (Figure 6B) of ‘Features’ we can deduce that dark proteins are located essentially in the extension of ‘Transmembrane’ regions (‘Helical’) and, like in Arabidopsis, are also ‘Signal’ sequences. They are under-represented in “Disolfides”, “Helix”, “Binding”, and “NP-bind” (Figure 10B).

E. Coli contains 32.5% of dark matter in its proteome. Attending TreeMap (Figure 7A) of ‘Descriptions’ of E. Coli, dark proteins are over-represented in larger number at the ‘Cell outer membrane’ mainly as a ‘Lipid anchor’. However, they are over-represented also at ‘Cell inner membrane’ where they are ‘Secreted’ in a slighter less quantity. These dark proteins also interact with themselves to form ligaments. Functions associated with them, not surprisingly include the ‘Conjunctive DNA transfer (CDT) which is the unidirectional transfer of ssDNA plasmid from a donor to a recipient cell which is the central mechanism by which antibiotic resistance and virulence factors are propagated in bacterial populations’. Dark proteins can also be associated with ‘Lysis’ proteins. According to the ‘Features’ TreeMap (Figure 7B) of E. Coli, dark proteins are over-represented in “Transmembrane (Helical)” and are associated with “Lipid” bonds. They are under-represented in Helix (Figure 10C).

S. Cerevisiae has 40.1% of its proteome on the dark side, and there are not sufficient proteins to infer conclusions about it using Fisher-tests for ‘Descriptions’ and ‘Features’. 

The Mus Musculus have 50.2% of dark proteome. Concerning ‘Descriptions’ (Figure 8A), the dark proteins of the mouse organism are over-represented in ‘Membrane’ with ‘Multi-pass’ and ‘Single-pass’, ‘Golgi Apparatus membrane’, ‘Mithocondrion’ and ‘Endoplasmic Reticulum membrane’. Dark proteins are under-represented in ‘Cell membrane’. They have “Tissue Specificity” in the ‘Lower and middle cortical regions of the hair shaft in both developing and cycling hair’. Dark proteins also ‘Interact with hair keratin’ having the purpose or function of keeping hair strong. In the ‘Hair cortex, hair keratin intermediate filaments are embedded in an interfilamentous matrix, consisting of hair keratin-associated proteins (KRTAP), which are essential for the formation of a rigid and resistant hair shaft through their extensive disulfide bond cross-linking with abundant cysteine residues of hair keratins. The matrix proteins include the high-sulfur and high-glycine-tyrosine keratins’. Focusing on ‘Features’ (Figure 8B) dark proteins are over-represented in “Transmembrane”, “Coiled”, and “Compositional Bias”. The “Topological Domains” of dark proteins are ‘Cytoplasmic’, ‘Lumenal’, and ‘Extracellular’. Dark proteins are under-represented in “Disulfide”, “Biding”, “Metal”, and “Activity Sites” (Figure 10D).

The last organism studied in this work was the Homo Sapiens (Human) proteome. It was found that over half of it (51.7%) was dark (Figure 1). The results for human enrichment analysis ‘Description’ (Figure 9A) gives dark proteins over-representation at “Subcellular Location”, like ‘Membrane’ (‘Multi-pass’ and ‘Single-pass’) were they ‘Shuttles between nucleolus and cytoplasm’ being also ‘Secreted’. About “Tissue Specificity’ they are over-represented in ‘Testis’, ‘Testis (tumor tissues)’, ‘Melanoma’ and ‘Carcinoma (bladder and lungs)’. Concerning “Functions”, it can be observed that dark proteins are directly linked with ‘Tumorigenesis’, ‘Tumor antigens’, and ‘Retroviral replication’. In “Caution” as stated in Reference [5], although using Swiss-Prot partly addresses the possibility that dark proteins may actually be unrecognized long noncoding RNA or may arise from pseudogenes were evidence occurs for a small number of cases. For the human enrichment analysis ‘Features’ (Figure 9B), all significant results are shown in “Transmembrane”, “Coiled”, “Compositional bias”, and ‘Cleavage’. Dark proteins are under-represented in ‘Disulfide’ (Figure 10E). In conclusion, we can add for the human case that less was known about the function and subcellular location of dark proteins, 56% shorter ‘CC’ field; missing location data for 56% compared with 22% for non-dark proteins (Figure 10F). 

### 3.2. Autonomy 

**Figure 11:**
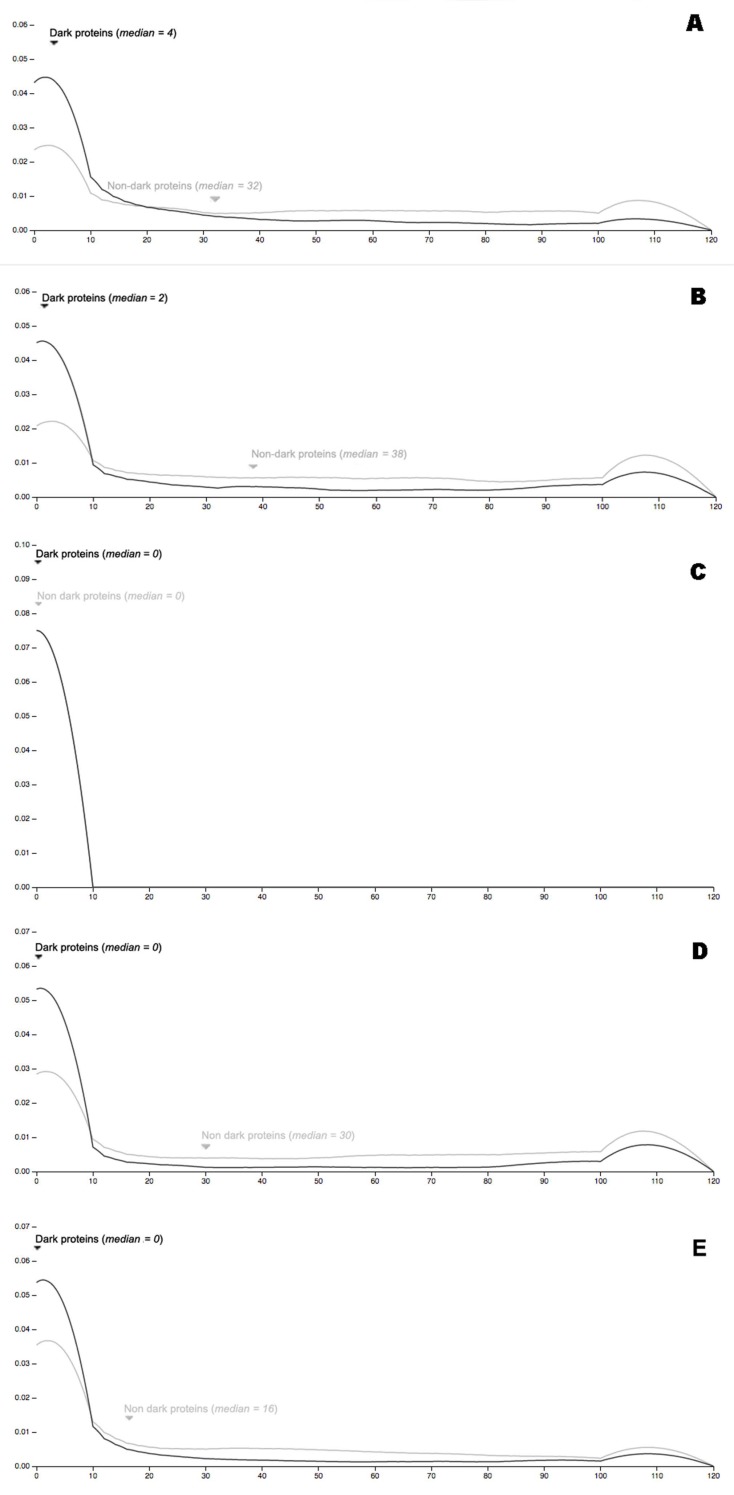
**(A)** Protein-proteins interactions (high quality = 700) for Arabidopsis using STRING; **(B)** Protein-proteins interactions (high quality = 700) for C. Elegans using STRING; **(C)** protein-proteins interactions (high quality = 700) for E. Coli using STRING; **(D)** protein-proteins interactions (high quality = 700) for Mouse using STRING; **(E)** protein-proteins interactions (high quality = 700) for Human using STRING.

Concerning autonomy in Arabidopsis (Figure 11A) it can be observed that dark proteins have much less interactions in comparison with non-dark proteins, which are quite high. Note the small peaks shown near 110 interactions that are a consequence of ribosomal proteins, these peaks will be present in all organisms (except E. Coli) below. The autonomy for C. Elegans follows the same previous pattern of Arabidopsis (Figure 11B), where dark proteins have much less interactions in comparison with non-dark proteins, that are fewer in comparison, but yet quite high. The autonomy for E. Coli has a curious result by comparing it with the previous cases (Figure 11C), where dark and non-dark proteins have the same number of interactions for high quality (700). The results for Yeast are similar with E. Coli. Concerning the Mouse autonomy (Figure 11D) it can be observed that dark proteins have fewer interactions in comparison with non-dark proteins of Mouse which are quite sound. Autonomy in the Human organism follows the same pattern presented in Mouse (Figure 11E), where dark proteins have much less interactions in comparison with non-dark proteins, however less than in Mouse organism.

### 3.3. Dark Genes 

It was also determined which dark proteins came from sequential genes, finding seven ‘dark’ gene clusters. Basically, you can take each protein and mapped down to the gene where the protein comes from and mapping down to chromosomes (See Methods), and if we do that proteins from these clusters had many features described above as typical for dark proteins (Table 5).

Length indicates the number of amino acids; ‘Binds’ indicates the number of known binding partners in the same cluster from STRING [10]; ‘Bias’ indicates the largest single amino acid composition (e.g., a value of ‘42%’ indicates that one amino acid accounts for 42% of the entire sequence) – the most frequently occurring amino acids are given for each cluster (e.g., ‘CS-rich’ indicates Cys is the most common, followed by Ser). The proteins arising from these gene clusters exhibit typical characteristics of dark proteins: they tend to be short, have few known interactions, have atypical amino acid composition, and are often secreted, transmembrane, or skin-associated. The 1q21.3 cluster arises from gene duplication [20]; it contains many skin proteins with significant compositional bias. The 4q13.3 cluster does not appear to have been previously characterized; it contains proteins related to the mouth, salivary glands, and secretion, implying that these genes share related functions. The 11q12 cluster arises from gene duplication during vertebrate evolution [18]; it contains proteins that all have a 4-pass membrane-spanning region and are components of a multimeric receptor complexes. The 17q21.2 and 21q22.11 clusters have also been previously identified [21,22]; they contain hair-associated proteins. The Xp11.23 and Xp11.22 clusters are both very recent evolutionary developments [23]; they contain proteins that are expressed only in testis and in cancer - some are also unique to human

### 3.4. Dark Tissues 

Finally, using ProteomicsDB [18] we looked at all the proteins that were expressed in 69 tissues, where every tissue has a list of expressed proteins and a level of abundance. What we have done was inspect each tissue (for instance, the brain), and observe which proteins where highly expressed and which fraction of those proteins were dark, associated with a darkness value, not for each protein, but for each tissue. The tissue that has the highest level of darkness is the heart, which is very interesting, since it is the tissue that is associated with heart disease, one of the main cause of death in humans (Table 6).

## 4. Discussion

Our previous work [5] didn’t point out solutions, but it opened a new field to be explored. This study is complementary to [6] through the delivery of new information concerning darkness present in Homo Sapiens and in model organisms related with it pharmacologically. It can be concluded that the amount of dark proteome present in all of them is still high, whereas in higher eukaryotes like mouse and human, it is around 50%. The results presented above are consistent with previous works [6,20] since Arabidopsis dark proteins are mainly located in extracellular space, cellular membrane, and endoplasmic reticulum membranes. C. Elegans dark proteins are present again in cell membrane where they are secreted. In the E. Coli case, it is reconfirmed that they are present in inner and outer membrane. Finally, in higher eukaryotic organisms like mouse, we observed that dark proteins are located in endoplasmic reticulum and in mitochondrion membrane, which is consistent with the previous results that state that dark proteins are mostly over-represented in specific secretory tissues and exterior environments, being also related to cancer endogenous retroviral proteins in the human organism [6]. Therefore, it was shown that dark proteins are not uniformly distributed throughout the different areas of the cell in organisms, where their presence is more common in some regions than in others. There are a lot of them in membranes, cell membranes or associated with transmembrane regions and cleavage, but they are less common in cytoplasm, where many globular proteins perform their activity. 

Concerning functions, the results confirmed that dark proteins perform a wide spectrum of functions depending on the organism in question, being more focused in simpler organisms and wider in higher organisms [6]. Again, it was found that a vast amount of them is programmed to live outside the cell, where many are associated with secretion (through secretory glands and ducts) or with extracellular areas in tissues, being an indicator that they possibly are designed for being defensive agents against external threats such as bacteria and/or virus. But we also observed that some of these dark proteins are subject to post-translational modifications, therefore being chemically modified after translation be applied.

Concerning autonomy, up to now there is no comprehensive map of all relevant functionally for PPI’s in simple or complex organisms. The existence of this map is of crucial importance to understand cellular behavior. Several databases started to flourish helping in the construction of this global protein interactions map. Some databases are dedicated to register interaction experiments such as physical binding detection among proteins [24,25,26,27]; others are centered on specific model organisms [28,29] However, there are two difficulties: The first is the “tsunami” of genome and proteome sequencing information that must be processed putting the above map in standby; The second difficulty is in the way proteins interact i.e., they also interact through indirect associations such as shared pathways which are not registered in interaction databases, but instead are registered in pathway databases [30,31,32] This is our contribution to the above map, especially to its dark side. The results show clear evidence that—independently of the organism evaluated—dark proteins have significantly fewer interactions with other proteins, in comparison with non-dark proteins. In general, we can conclude that dark proteins are more independent and autonomous than non-dark proteins. Therefore, the DPD is a map for the dark proteome at the present time where the model organisms described are already available together with its functional analysis, augmenting the knowledge about them, where we have work in progress for all the remaining organisms. 

**Figure 12:**
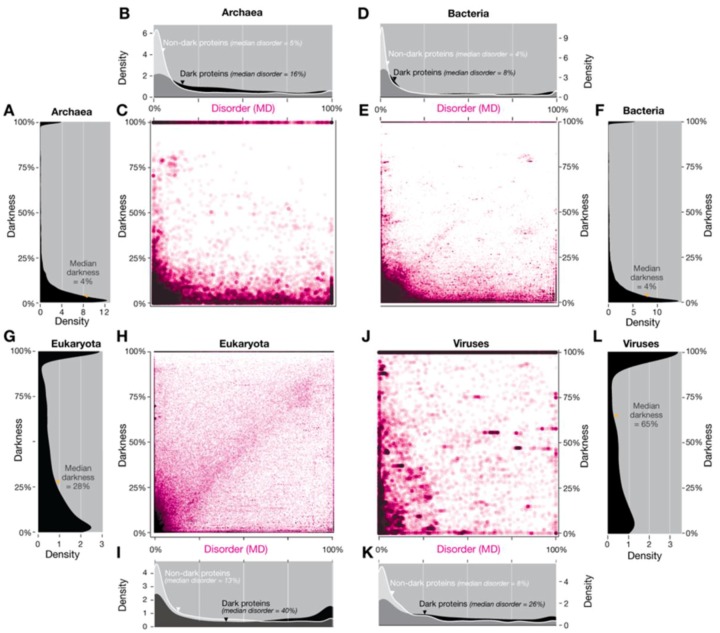
Comparing darkness with disorder defined using MD applied to the four domains of life. (**A**) The distribution of darkness in Archaea; (**B**) The distribution of disorder in Archaea; (**C**) Relation between darkness and disorder in Archaea; (**D**) The distribution of disorder in Bacteria (**E**) Relation between darkness and disorder in Bacteria (**F**) The distribution of darkness in Bacteria; (**G**) The distribution of darkness in Eukaryota; (**H**) Relation between darkness and disorder in Eukaryota (**I)** The distribution of disorder in Eukaryota; (**J**) Relation between darkness and disorder in Viruses; (**K**) The distribution of disorder in Viruses; (**L**) The distribution of darkness in Viruses. Overall, the results are mostly similar to those obtained using only IUPred (Figure 2 and Figure S3 of Reference [5]). For eukaryotes, however, using MD results in a larger fraction of proteins occur close to the diagonal, resulting in an approximately linear relationship between disorder and darkness (H), in contrast to the upper triangular region seen with IUPred (Figure 2C of Reference [5]). However, as previously, most proteins do not show this trend. Indeed, the presence of almost as many proteins below this region as above indicates that disorder is essentially unrelated to darkness. For viruses (J), the pattern associated with disordered linear motifs is even more pronounced (Figure S3C of Reference [5]). The density plots (B, D, I, and K) show that MD disorder is more evenly distributed than IUPred disorder (Figure 2 and Figure S3 of Reference [5], respectively).

A point that we want to bring to discussion is the difference between intrinsically disordered proteins (IDP’s) and their relationship with the Dark Proteome (DP) as we coined it in 2015 [5], where we concluded that the Dark Proteome is mostly not disordered using the predictor IUPred [33]. The need to use a predictor was because only 62 proteins of Swiss-Prot (data from 2014) existed with ´disordered´ annotations from a total of 546,000 proteins. In our subsequent work [6] (data from 2016), the same 62 proteins remained but now among a set of 550,116 Swiss-Prot proteins. For this work, considering ‘disordered’ annotations for the Human organism we would have (data from 2016 [6]) only one protein to work with: Q8WYP5 (the same for 2014 data [5]). Hence, we make the hypothesis: If we use another predictor do we get a different result? The answer is yes, but no. The disorder values shown in Figure 2 and Figure S3 of Perdigão et al. [5] were calculated using IUPred [33] because it is one of the most widely used methods for predicting disorder. Residues were defined as disordered if they had an IUPred score ≥0.5 [5]. In this study, we also calculated a second set of disorder values using MD (META-Disorder) [34], a machine-learning method that calculates a consensus disorder from several orthogonal methods. Re-plotting the density and scatterplots Figure 2 and Figure S3 of Reference [5] using MD disorder gave a similar overall pattern, although some differences were apparent (Figure 12). MD includes as one of its input methods DISOPRED2 [35], which is one of several available methods that are optimized to predict residues missing from PDB structures. For a small fraction of proteins there were not MD predictions; to balance the comparisons, these proteins were removed from the density and scatterplots in Figure 2 and Figure S3 of Reference [5]—thus reducing the number of proteins to 175.646 in archaea, 18.999 in bacteria, 326.945 in eukaryotes and 16.316 in viruses, respectively.

Intrinsic disorder in proteins is a complex and poorly understood phenomenon, in addition to IUPred, many other prediction methods have been developed focusing on a range of different aspects of disorder [36]. It would certainly be of interest to compare darkness with disorder predictions from a range of methods, however the output from these algorithms is difficult to decode due to the lack of metrics or references to compare with [37]. The DPD wants to help in a near future with the introduction of three new disorder predictors applied in the Swiss-Prot universe.

## 5. Conclusions

Five hundred years ago, very little of the Earth was known. People suspect that it was a sphere, with land and water and they had roughly mapped out Europe, but that was it. Knowing what they didn’t know gave Portuguese explorers like Pedro Álvares Cabral, Vasco da Gama, and Fernão de Magalhães a direction in which to head—the same principle applies to science and discovery today. We have been able to identify regions within each protein that are different to any region where the structure has been determined experimentally. This unknown area is called the ‘dark proteome’ and actually accounts for nearly half the proteins in viruses and in eukaryotes, which includes humans. It will provide insight into protein-based illnesses like cancer, type 2 Diabetes, and many neurodegenerative diseases, such as Parkinson’s and Alzheimer’s. Just like the early Portuguese explorers that discovered Africa, America, and Asia, knowing what we don’t know has provided us with a roadmap upon which to focus our future research and agendas. Knowing that the Dark Proteome is mostly not disordered, mostly not compositionally biased, mostly not transmembrane and that dark proteins are mostly Unknown Unknowns, the purpose of this study was a detailed characterization of the dark proteins belonging to the human and model organisms under the pharmacological umbrella. Because we already saw too many unexpected surprises, the next step of DPD are the IDP’s because who knows what secrets are still hidden in the dark proteome. As far as we are concerned, we are only interested in the truth, regardless of how unexpected, difficult or amazing it will be.

## Figures and Tables

**Table 1:** Annotations enriched (Features field - FT) in dark proteins from eukaryota (only the first 20 entries).

Non-dark	Dark	Ratio	Total	Fisher’s p value	Adjusted p value	Annotation sub-category	Annotation
1	380	2397.28	381	0	0	SIMILARITY	Belongs to the Casparian strip membrane proteins (CASP) family. {ECO:0000305}.
1722	1900	6.96	3622	0	0	SUBCELLULAR_LOCATION	Membrane {ECO:0000305}; Multi-pass membrane protein {ECO:0000305}.
182	744	25.79	926	0	0	TISSUE_SPECIFICITY	Expressed by the venom duct.
1	376	2372.04	377	2.96 × 10^–323^	2.31 × 10^–318^	SUBUNIT	Homodimer and heterodimers. {ECO:0000250}.
966	900	5.878	1866	2.94 × 10^–281^	1.84 × 10^–276^	SUBCELLULAR_LOCATION	Membrane {ECO:0000305}; Singl × 10–pass membrane protein {ECO:0000305}.
14	276	124.37	290	9.18 × 10^–217^	4.77 × 10^–212^	DOMAIN	The presence of a ’disulfide through disulfide knot’ structurally defines this protein as a knottin. {ECO:0000250}.
448	557	7.84	1005	1.80 × 10^–212^	8.02 × 10^–208^	SUBCELLULAR_LOCATION	Cell membrane {ECO:0000250}; Multi-pass membrane protein {ECO:0000250}.
39	250	40.44	289	9.16 × 10^–171^	3.57 × 10^–166^	TISSUE_SPECIFICITY	Testis.
0	196	0	196	4.22 × 10^–170^	1.46 × 10^–165^	SIMILARITY	Belongs to the PsbN family. {ECO:0000255|HAMAP-Rule:MF_00293}.
0	194	0	194	2.26 × 10^–168^	7.06 × 10^–164^	FUNCTION	May play a role in photosystem I and II biogenesis. {ECO:0000255|HAMAP-Rule:MF_00293}.
0	189	0	189	4.75 × 10^–164^	1.35 × 10^–159^	SUBCELLULAR_LOCATION	Plastid, chloroplast thylakoid membrane {ECO:0000255|HAMAP-Rule:MF_00293}; Single-pass membrane protein {ECO:0000255|HAMAP-Rule:MF_00293}.
412	456	6.98	868	6.67 × 10^–162^	1.73 × 10^–157^	SUBCELLULAR_LOCATION	Endoplasmic reticulum membrane {ECO:0000250}; Multi-pass membrane protein {ECO:0000250}.
0	184	0	184	9.98 × 10^–160^	2.39 × 10^–155^	CAUTION	Originally thought to be a component of PSII; based on experiments in Synechocystis, N.tabacum and barley, and its absence from PSII in T.elongatus and T.vulcanus, this is probably not true. {ECO:0000255
26	217	52.65	243	4.35 × 10^–155^	9.69 × 10^–151^	SIMILARITY	Belongs to the conotoxin O1 superfamily. {ECO:0000305}.
87	258	18.71	345	6.35 × 10^–146^	1.32 × 10^–141^	SIMILARITY	Belongs to the DEFL family. {ECO:0000305}.
1	155	977.84	156	1.57 × 10^–132^	3.06 × 10^–128^	SIMILARITY	Belongs to the protamine P1 family. {ECO:0000305}.
0	145	0	145	5.12 × 10^–126^	9.40 × 10^–122^	FUNCTION	Mitochondrial membrane ATP synthase (F(1)F(0) ATP synthase or Complex V) produces ATP from ADP in the presence of a proton gradient across the membrane which is generated by electron transport complexes of the respiratory chain. F-type ATPases consist of two structural domains, F(1) - containing the extramembraneous catalytic core and F(0) - containing the membrane proton channel, linked together by a central stalk and a peripheral stalk. During catalysis, ATP synthesis in the catalytic domain of F(1) is coupled via a rotary mechanism of the central stalk subunits to proton translocation. Part of the complex F(0) domain. Minor subunit located with subunit a in the membrane (By similarity). {ECO:0000250}.
0	143	0	143	2.74 × 10^–124^	4.75 × 10^–120^	SUBCELLULAR_LOCATION	Mitochondrion membrane; Singl × 10–pass membrane protein.
0	142	0	142	2.01 × 10^–123^	3.29 × 10^–119^	SIMILARITY	Belongs to the ATPase protein 8 family. {ECO:0000305}.
2313	18	0.05	2331	3.33 × 10^–119^	5.20 × 10^–115^	CATALYTIC_ACTIVITY	ATP + a protein = ADP + a phosphoprotein.

**Table 2:** Annotations enriched (Descriptions field – DE) in dark proteins from eukaryota (only the first 20 entries).

Non-dark	Dark	Ratio	Total	Fisher’s p value	Adjusted p value	Annotation sub-category	Annotation
96,211	24,496	3.25	120,707	0	0	transmem	Helical; (Potential)
15,723	3753	3.05	19,476	0	0	signal	Potential
45	562	159.48	607	0	0	mod_res	Phenylalanine amide
4152	1677	5.16	5829	0	0	topo_dom	Lumenal (Potential)
69	524	96.98	593	0	0	mod_res	Leucine amide
35,139	6509	2.37	41,648	0	0	topo_dom	Cytoplasmic (Potential)
16,045	11	0.01	16,056	0	0	binding	Substrate (By similarity)
6648	3000	5.76	9648	0	0	non_ter	
10,317	2823	3.49	13,140	0	0	coiled	Potential
110,128	0	0	110,128	0	0	strand	
27,108	0	0	27,108	0	0	turn	
103,913	0	0	103,913	0	0	helix	
44	312	90.55	356	5.19 × 10^–301^	1.79 × 10^–296^	mod_res	Valine amide
8891	1	0	8892	1.69 × 10^–289^	5.43 × 10^–285^	np_bind	ATP (By similarity)
24,535	3794	1.97	28,329	2.95 × 10^–287^	8.82 × 10^–283^	mod_res	Phosphoserine (By similarity)
904	596	8.42	1500	1.95 × 10^–273^	5.46 × 10^–269^	unsure	L or I
8122	2	0	8124	8.37 × 10^–262^	2.21 × 10^–257^	act_site	Proton acceptor (By similarity)
1186	641	6.90	1827	1.49 × 10^–257^	3.70 × 10^–253^	non_cons	
401	414	13.18	815	8.24 × 10^–242^	1.94 × 10^–237^	mod_res	Pyrrolidone carboxylic acid
6967	0	0	6967	5.48 × 10^–229^	1.23 × 10^–224^	np_bind	GTP (By similarity)

**Table 3:** Annotations enriched (Description field - DE) in dark proteins from Homo Sapiens (only the first 20 entries).

Non-dark	Dark	Ratio	Total	Fisher’s p value	Adjusted p value	Annotation sub-category	Annotation
124	28	18.49	152	9.71 × 10^–25^	6.65 × 10^–20^	SUBCELLULAR_LOCATION	Membrane; Single-pass membrane protein (Potential).
0	11	0	11	7.55 × 10^–22^	2.59 × 10^–17^	CAUTION	Product of a dubious CDS prediction. May be a non-coding RNA.
162	27	13.64	189	7.11 × 10^–21^	1.62 × 10^–16^	CAUTION	Could be the product of a pseudogene.
5	12	196.47	17	5.29 × 10^–20^	9.05 × 10^–16^	CAUTION	Product of a dubious CDS prediction.
0	9	0	9	5.27 × 10^–18^	7.22 × 10^–14^	MISCELLANEOUS	Encoded by one of the numerous copies of NBPF genes clustered in the p36, p12 and q21 region of the chromosome 1.
0	9	0	9	5.27 × 10^–18^	6.01 × 10^–14^	SIMILARITY	Belongs to the beta type-B retroviral envelope protein family. HERV class-II K(HML-2) env subfamily.
0	8	0	8	4.39 × 10^–16^	4.30 × 10^–12^	FUNCTION	Retroviral replication requires the nuclear export and translation of unspliced, singly-spliced and multiply-spliced derivatives of the initial genomic transcript. Rec interacts with a highly structured RNA element (RcRE) present in the viral 3’LTR and recruits the cellular nuclear export machinery. This permits export to the cytoplasm of unspliced genomic or incompletely spliced subgenomic viral transcripts (By similarity).
0	8	0	8	4.39 × 10^–16^	3.76 × 10^–12^	SUBUNIT	Forms homodimers, homotrimers, and homotetramers via a C-terminal domain. Associates with XPO1 and with ZNF145 (By similarity).
1	8	654.91	9	3.91 × 10^–15^	2.98 × 10^–11^	PTM	Specific enzymatic cleavages in vivo yield the mature SU and TM proteins (By similarity).
0	7	0	7	3.66 × 10^–14^	2.51 × 10^–10^	SUBCELLULAR_LOCATION	Cytoplasm (By similarity). Nucleus, nucleolus (By similarity). Note=Shuttles between the nucleus and the cytoplasm. When in the nucleus, resides in the nucleolus (By similarity).
3	8	218.30	11	7.02 × 10^–14^	4.37 × 10^–10^	SUBUNIT	The surface (SU) and transmembrane (TM) proteins form a heterodimer. SU and TM are attached by noncovalent interactions or by a labile interchain disulfide bond (By similarity).
218	21	7.89	239	2.55 × 10^–12^	1.46 × 10^–08^	SUBCELLULAR_LOCATION	Membrane; Multi-pass membrane protein (Potential).
0	5	0	5	2.54 × 10^–10^	1.34 × 10^–06^	MISCELLANEOUS	The ancestral BAGE gene was generated by juxtacentromeric reshuffling of the KMT2C/MLL3 gene. The BAGE family was expanded by juxtacentromeric movement and/or acrocentric exchanges. BAGE family is composed of expressed genes that map to the juxtacentromeric regions of chromosomes 13 and 21 and of unexpressed gene fragments that scattered in the juxtacentromeric regions of several chromosomes, including chromosomes 9, 13, 18 and 21.
0	5	0	5	2.54 × 10^–10^	1.24 × 10^–06^	SIMILARITY	Belongs to the BAGE family.
0	5	0	5	2.54 × 10^–10^	1.16 × 10^–06^	CAUTION	Product of a dubious CDS prediction. Probable non-coding RNA.
215	17	6.47	232	4.87 × 10^–09^	2.09 × 10^–05^	SUBCELLULAR_LOCATION	Secreted (Potential).
2	5	204.66	7	5.22 × 10^–09^	2.10 × 10^–05^	FUNCTION	Retroviral envelope proteins mediate receptor recognition and membrane fusion during early infection. Endogenous envelope proteins may have kept, lost or modified their original function during evolution. This endogenous envelope protein has lost its original fusogenic properties.
3	5	136.44	8	1.38 × 10^–08^	5.25 × 10^–05^	SUBUNIT	Complex I is composed of 45 different subunits.
0	4	0	4	2.11 × 10^–08^	7.61 × 10^–05^	CAUTION	No predictable signal peptide.
0	4	0	4	2.11 × 10^–08^	7.23 × 10^–05^	SIMILARITY	Belongs to the Speedy/Ringo family.

**Table 4:** Annotations enriched (Features field - FT) in dark proteins from Homo Sapiens (only the first 20 entries).

Non-dark	Dark	Ratio	Total	Fisher’s p value	Adjusted p value	Annotation sub-category	Annotation
50,327	0	0	50,327	3.58 × 10^–140^	5.65 × 10^–135^	strand	
46,207	0	0	46,207	6.54 × 10^–128^	5.15 × 10^–123^	helix	
7572	217	4.75	7789	2.33 × 10^–76^	1.22 × 10^–71^	transmem	Helical; (Potential)
2023	81	6.64	2104	1.29 × 10^–38^	5.10 × 10^–34^	coiled	Potential
11,972	0	0	11,972	3.59 × 10^–32^	1.13 × 10^–27^	turn	
12,033	5	0.07	12,038	4.92 × 10^–25^	1.29 × 10^–20^	disulfid	By similarity
0	8	0	8	1.65 × 10^–18^	3.71 × 10^–14^	domain	NBPF 3
0	8	0	8	1.65 × 10^–18^	3.24 × 10^–14^	domain	NBPF 1
0	8	0	8	1.65 × 10^–18^	2.88 × 10^–14^	domain	NBPF 2
152	19	20.73	171	1.84 × 10^–18^	2.89 × 10^–14^	compbias	Poly-Arg
3	9	497.59	12	2.13 × 10^–18^	3.05 × 10^–14^	motif	Nuclear export signal (Potential)
3	8	442.30	11	2.67 × 10^–16^	3.51 × 10^–12^	region	Fusion peptide (Potential)
0	7	0	7	2.75 × 10^–16^	3.34 × 10^–12^	domain	NBPF 4
0	7	0	7	2.75 × 10^–16^	3.10 × 10^–12^	domain	NBPF 5
1939	46	3.93	1985	2.92 × 10^–14^	3.07 × 10^–10^	signal	Potential
0	6	0	6	4.61 × 10^–14^	4.54 × 10^–10^	domain	NBPF 6
482	20	6.88	502	6.27 × 10^–11^	5.81 × 10^–7^	compbias	Poly-Glu
33	8	40.20	41	1.32 × 10^–10^	1.16 × 10^–6^	site	Cleavage (By similarity)
0	4	0	4	1.29 × 10^–9^	1.07 × 10^–5^	mutagen	C->S: No significant activity
3356	0	0	3356	3.16 × 10^–9^	2.49 × 10^–5^	disulfid	

**Table 5:** Human gene clusters containing dark proteins.

Gene	Protein	Length	Binds	Bias	Gene	Protein	Length	Binds	Bias
*Chromosome 1 (q21.3): PQCK-rich, keratinocyte proteins*					*Chromosome 17 (q21.2): CS-rich, keratin associated proteins*				
LCE5A	Late cornified envelope 5A	118	3	21	KRTAP3-3	Keratin associated protein 3-3	98		19
CRCT1	Cysteine-rich C-terminal 1	99	2	25	KRTAP3-2	Keratin associated protein 3-2	98		19
LCE3E	Late cornified envelope 3E	92	2	16	KRTAP3-1	Keratin associated protein 3-1	98		18
LCE3E	Late cornified envelope 3E	92	2	17	KRTAP3-1	Keratin associated protein 3-1	98		24
LCE3D	Late cornified envelope 3D	92	2	19	KRTAP1-5	Keratin associated protein 1-5	174		25
LCE3C	Late cornified envelope 3C	94	4	19	KRTAP1-1	Keratin associated protein 1-1	177		27
LCE3B	Late cornified envelope 3B	95	2	24	KRTAP2-1	Keratin associated protein 2-1	128		27
LCE3A	Late cornified envelope 3A	89	1	21	KRTAP2-1	Keratin associated protein 2-1	128		35
LCE2D	Late cornified envelope 2D	110	1	20	KRTAP4-11	Keratin associated protein 4-11	195		37
LCE2C	Late cornified envelope 2C	110		21	KRTAP4-12	Keratin associated protein 4-12	201		37
LCE2B	Late cornified envelope 2B	110		22	KRTAP4-4	Keratin associated protein 4-4	166		36
LCE2A	Late cornified envelope 2A	106	1	20	KRTAP4-3	Keratin associated protein 4-3	195		35
LCE4A	Late cornified envelope 4A	99	3	18	KRTAP4-2	Keratin associated protein 4-2	136		34
KPRP	Keratinocyte proline-rich protein	579	1	20	KRTAP4-1	Keratin associated protein 4-1	146		36
LCE1F	Late cornified envelope 1F	118		22	KRTAP17-1	Keratin associated protein 17-1	105		19
LCE1E	Late cornified envelope 1E	118	1	22	*Chromosome 21 (q22.11): GYSC-rich, keratin-associated proteins*				
LCE1D	Late cornified envelope 1D	114	1	22	CLDN17	Claudin 17	224	1	14
LCE1C	Late cornified envelope 1C	118	1	21	CLDN8	Claudin 8	225	1	10
LCE1B	Late cornified envelope 1B	118		20	KRTAP24-1	Keratin associated protein 24-1	254	2	19
LCE1A	Late cornified envelope 1A	110	1	14	KRTAP25-1	Keratin associated protein 25-1	102		21
LCE6A	Late cornified envelope 6A	80		17	KRTAP26-1	Keratin associated protein 26-1	210	2	18
SMCP	Sperm mitochondria-associated	116		29	KRTAP27-1	Keratin associated protein 27-1	207	2	16
SPRR4	Small proline-rich protein 4	79		22	KRTAP23-1	Keratin associated protein 23-1	65	2	20
SPRR3	Small proline-rich protein 3	169		29	KRTAP13-2	Keratin associated protein 13-6, pseudogene	175		23
SPRR1B	Small proline-rich protein 1B	89		38	KRTAP13-1	Keratin associated protein 13-1	172		23
SPRR2D	Small proline-rich protein 2D	72		38	KRTAP13-3	Keratin associated protein 13-3	172		22
SPRR2A	Small proline-rich protein 2A	72	1	39	KRTAP13-4	Keratin associated protein 13-4	160		21
SPRR2B	Small proline-rich protein 2B	72	1	39	KRTAP19-1	Keratin associated protein 19-1	90		42
SPRR2E	Small proline-rich protein 2E	72		36	KRTAP19-2	Keratin associated protein 19-2	52		27
SPRR2F	Small proline-rich protein 2F	72		40	KRTAP19-3	Keratin associated protein 19-3	81		43
SPRR2G	Small proline-rich protein 2G	73		26	KRTAP19-4	Keratin associated protein 19-4	84		27
LELP1	Late cornified envelope-like	98		21	KRTAP19-5	Keratin associated protein 19-5	72		39
*Chromosome 4 (q13.3): P-rich, mouth and digestive secreted proteins*					KRTAP19-7	Keratin associated protein 19-7	63	2	33
CSN1S1	Casein alpha s1	185	2	11	KRTAP6-2	Keratin associated protein 6-2	62		32
CSN2	Casein beta	226	2	17	KRTAP6-1	Keratin associated protein 6-1	71		38
STATH	Statherin	62	2	11	KRTAP20-1	Keratin associated protein 20-1	56		36
HTN3	Histatin 3	51	1	14	KRTAP20-2	Keratin associated protein 20-2	65		37
HTN1	Histatin 1	57	2	12	KRTAP20-3	Keratin associated protein 20-3	44	4	25
C4orf40	Proline-rich protein 27	219		21	KRTAP21-1	Keratin associated protein 21-1	79	2	35
ODAM	Odontogenic, ameloblast asssociated	279		15	KRTAP8-1	Keratin associated protein 8-1	63		24
C4orf7	Follicular dendritic cell secreted	85		19	KRTAP11-1	Keratin associated protein 11-1	163		15
CSN3	Casein kappa	182	2	16	KRTAP19-8	Keratin associated protein 19-8	63	4	35
SMR3B	Salivary gland androgen regulated	79	1	39	*Chromosome X (p11.23): EPG-rich, GAGE and PAGE family* proteins				
MUC7	Mucin 7, secreted	377	4	20	GAGE10	G antigen 10	116		17
AMTN	Amelotin	209	1	15	GAGE12J	G antigen 12J	117		16
AMBN	Enamel matrix protein	447	1	15	GAGE12F	G antigen 6	117		17
IGJ	Immunoglobulin J chain	159	1	9	GAGE13	G antigen 13	117		17
UTP3	Processome component	479	1	13	GAGE2E	G antigen 8	116		17
*Chromosome 11 (q12.1-q12.2): LS-rich, transmembrane complex members*					GAGE2D	G antigen 8	116		16
MS4A3	Member 3	214		13	GAGE2C	G antigen 2C	116		18
MS4A2	Member 2, receptor for	244		12	GAGE12B	G antigen 12B	117		17
MS4A6A	Member 6A	248	2	14	GAGE2A	G antigen 2A	116		17
MS4A4E	Putative member 4E	132	2	11	GAGE1	G antigen 6	139		14
MS4A4A	Member 4	239	1	11	GAGE4	Cancer/testis antigen 4.4	117		17
MS4A6E	Member 6E	147	2	16	PAGE1	P antigen family, member 1	146		18
MS4A7	Member 7	240	1	15	PAGE4	P antigen family, member 4	102		15
MS4A5	Member 5	200		13	*Chromosome X (p11.22): EP-rich; contains XAGE family proteins*				
					XAGE2B	X antigen family, member 2B	111		13
					XAGE1B	G antigen member; Cancer/testis antigen 12.1	81		15
					SSX7	Synovial sarcoma, X breakpoint 7	188		12
					SSX2B	Synovial sarcoma, X breakpoint 2B	188		12
					SPANXN5	SPANX family, member N5	72		14
					XAGE5	X antigen family, member 5	108		12
					XAGE3	X antigen family, member 3	111		15
					FAM156A	Family with sequence similarity 156, member B	213		12

**Table 6:** Tissues with the highest levels of darkness (only the first 25 entries).

Rank	Tissue	Ratio Dark Residues
1	Heart	50%
2	Cervical Mucosa	50%
3	Natural Killer Cell	50%
4	Lung	49%
5	Testis	49%
6	Rectum	49%
7	Proximal Fluid Coronary Sinus	49%
8	Pancreas	49%
9	B. Lymphocyte	49%
10	Colon Muscle	49%
11	Bone Marrow Stromal Cell	48%
12	Hair Follicle	48%
13	Cytotoxic T Lymphocyte	48%
14	Helper T Lymphocyte	48%
15	Colon	48%
16	Ovary	48%
17	Stomach	48%
18	Spinal Cord	47%
19	Placenta	47%
20	Vitreous Humor	47%
21	Blood Platelet	47%
22	Prostate Gland	47%
23	Retina	47%
24	Salivary Gland	47%
25	Uterus	46%

## Supplementary Materials

The datasets generated during and/or analyzed during the current study will be available in the Dark Proteome Database site [http://www.darkproteome.ws/darkproteins].
