# Supplementary figures and images for: Dark Proteome Database: Studies on Dark Proteins
Source: High Throughput. 2019 Mar 27;8(2):8. doi: 10.3390/ht8020008 (PMC6630768; doi:10.3390/ht8020008)

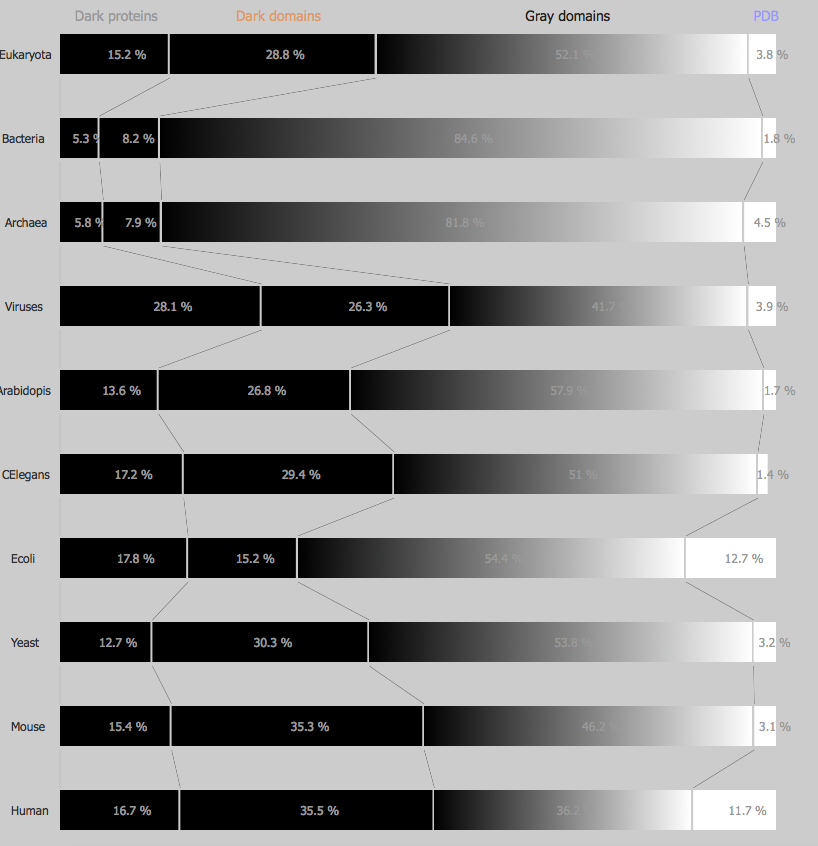

Supplement: Supplementary file 1 [file high-throughput-08-00008-s001.zip › high-throughput-409103-suppl-final/Figure S1.png]
